# Nutritional and sensory properties: Snack food made from high‐quality cassava flour and legume blend

**DOI:** 10.1002/fsn3.464

**Published:** 2017-03-01

**Authors:** Busie Maziya‐Dixon, Emmanuel O. Alamu, Ibironke O. Popoola, Marie Yomeni

**Affiliations:** ^1^ Food and Nutrition Sciences Laboratory, International Institute of Tropical Agriculture (IITA)IbadanNigeria; ^2^ Food and Nutrition Sciences LaboratoryInternational Institute of Tropical Agriculture(IITA)Southern Africa Research and Administration Hub (SARAH) CampusChelstonLusakaZambia; ^3^ International Institute of Tropical AgricultureBukavuDemocratic Republic Congo

**Keywords:** Antinutrients, cassava flour, consumer acceptance, snack foods

## Abstract

The nutritional benefits of grain legumes such as cowpea and soybean in sub‐Saharan Africa have not been fully utilized to alleviate problem of protein‐malnutrition in this region. This study aimed to evaluate and compare the nutritional properties and sensory evaluation of snack food made from high‐quality cassava flour (HQCF) and soybeans (50:50), and HQCF and cowpea (50:50). Sensory evaluation was conducted among panellists in Nigeria and DR Congo. Results showed that the soy variant of the snack contained significantly higher protein than the cowpea variant. There was cross‐cultural difference in snack preference between panellists in Nigeria and DR Congo. Panellists in DR Congo preferred the aroma of the unboiled soy variant, whereas panellists in Nigeria preferred the boiled soy variant more. This study showed the potential of legumes and cassava in the snack food sector not only as a partial substitute for wheat flour but as a major ingredient and also form basis for the new product development in the snacks food industry. The developed product could be possibly used to alleviate the protein‐malnutrition among the vulnerable groups of most developing countries.

## 1 Introduction

Urbanization has been a major contributing factor to changes in food consumption and dietary patterns for many people (Popkin, 1999) including consumers in developing countries. Owing to the fast‐paced nature of urban living, there is a high dependence on convenience and snack foods among urban dwellers. With an urbanization rate of over 4% (Kearney, 2010), most sub‐Saharan African countries are not left out of the convenience food trend. The majority of these convenience foods (e.g., biscuit, burgers, croissants) are made principally from wheat flour which is not abundant in most sub‐Saharan countries due to the tropical weather in these countries which is unfavorable for wheat cultivation. Hence, there is huge reliance on imported wheat to meet the demand for convenience foods for the ever increasing urban population (Oluwamukomi, Oluwalana, & Akinbowale, 2011). On the other hand, tropical root crops which are abundant in these countries are mainly utilized as staples with a large proportion lost at postharvest stage. An example of such a tropical root crop is cassava. Africa accounted for over 50% of cassava production in the world in 2007 (Aniedu & Omodamiro, 2012) and cassava production is projected to expand in this continent (FAO 2013). Of the 228 million tons of cassava produced in 2007, Nigeria alone accounted for 46 million tons making it the world's largest producer of the crop. On the other hand, the Democratic Republic of Congo (DRC) is the fifth largest producer after Nigeria, Brazil, Thailand, and Indonesia but the majority of cassava is utilized as flour made into dough (Phillips, Taylor, Sanni, & Akoroda, 2004).

The high cost of wheat importation is gradually making confectionery rather expensive, so calling for the development of suitable alternatives for wheat flour. Studies have identified cassava as a suitable partial replacement for wheat flour due to its high yield per hectare, low cost of production (Ogunjobi & Ogunwolu, 2010), ability to thrive under tropical conditions and poor soils as well as its limited land requirement for cultivation and its drought tolerance ability (Ohimain, 2014). High‐quality cassava flour (HQCF), that is, unfermented cassava flour described by Bokanga (1995), has been successfully used as a partial and complete replacement for wheat flour in bread, cookies, and other confectionery. The numerous benefits of cassava notwithstanding, cassava contains low levels of protein and thus has to be supplemented with protein‐rich foods such as legumes for it to be nutritious. Various studies have shown a decrease in protein levels of confectionery substituted with cassava flour, whereas studies have also attempted to make up for the reduced level of protein in composite cassava products by adding other protein‐rich food sources such as legume flour. Development of snack foods from HQCF that will be highly compared or surpassed those from 100% wheat flour in terms of protein and other nutrients have not been fully developed and exploited.

Grain legumes such as cowpea and soybean contain considerable amount of proteins and like cassava, their production is relatively high in sub‐Saharan Africa. Nigeria also accounts for a significant proportion of cowpea production in Africa (IITA 2009) and consumption is mainly limited to staples with relatively no utilization in convenience and snack foods. Similarly, soybean is an excellent source of good quality protein containing over 36% protein and is also relatively abundant in Nigeria. Nigeria is the largest producer of soybean in Africa; accounting for 39% of production followed by South Africa which accounted for 35% of soybean production in Africa (Adelodun, 2011) whereas DR Congo is the 8^th^ largest producer of soybean in Africa.

Increasing urbanization, changing dietary patterns, and increasing demand for convenience foods calls for the development of convenience foods that are both nutritious and appealing to the senses. There is need to develop such food products with little or no dependence on imported raw materials due to the already high cost of wheat importation. Cassava has limited application in confectionery and snack food production. Previous studies on the utilization of cassava in confectionery have been usually based on using cassava as a partial substitute for wheat flour, especially in bread production and biscuit production (Eggleston, Omoaka, & Ihedioha, 1992; Oluwamukomi et al., 2011), whereas there are few snack foods with cassava as major ingredient, that is, not as a partial substitute.

Cassava strips are a relatively new snack food made from a combination of HQCF and cowpea mixed in a ratio of 1:1 (Aniedu & Omodamiro, 2012; Sanni et al., 2006). The combination of ingredients makes it a complete, nutritious food. Consumption of this snack food is gradually gaining acceptance in Nigeria as small‐scale processors are already making the products for sale in supermarkets and even online stores. Although the original recipe was developed using cowpea or beans, the possibility of utilizing other protein‐rich legumes is worth exploring.

Soybean contains a considerably higher level of protein than other legumes and is known to contain isoflavones which are believed to have anticancer properties (Akaninwor & Okechukwu, 2004). The protein quality of soybean is also comparable to animal protein sources such as meat, poultry, and eggs as soy protein contains essential amino acids limited in other plant protein sources (Beski, Swick, & Iji, 2015). Development of the soybean variant of the snack food may provide a more nutritious alternative to the cowpea variant. This study thus examined the possibility of developing and promoting the soybean variant of this snack food by comparing nutritional, antinutritional, and sensory properties and consumer acceptance of both the cowpea and soybean variant of the snack. In spite of the nutritional benefits of soybean, it contains antinutritional factors which adversely affect the bioavailability and digestibility of its nutrients in levels considerably higher than other legumes. (Owolabi, Ndidi, James, & Amune, 2012) reported a tannin content of between 1.64 mg/100 g and 6.01 mg in improved and local varieties of cowpea, whereas soybean contained as much as 63.36 mg/100 g tannin (Megat‐Rusydi & Azrina, 2012). Studies have shown that the antinutritional factors in soybean are significantly reduced by soaking and boiling (Soetan & Oyewole, 2009). A study by (Kaankuka, Balogun, & Tegbe, 1996) showed a significant decrease in trypsin inhibitor activity and tannin levels with increasing cooking time while phytic acid reduced nonsignificantly. Hence, this study also aimed to investigate the effect of short time boiling of soybean before further processing on the nutritional, antinutritional, and sensory preferences of the snack food. This is necessary considering the presence of antinutritional factors in soybean which limit bioavailability of its nutrients and also the beany flavor synonymous with soybean that consumers find unacceptable in some soybean products, particularly soymilk. The findings of this study will demonstrate the potential application of cassava and grain legumes in the snack and confectionery food sector not only as a partial substitute for wheat flour but as major ingredients.

## 2 Methodology

### 2.1 Sample preparation

The strip samples were prepared using the method described by (Aniedu & Omodamiro, 2012; Sanni et al., 2006) with slight modifications. Samples were made using HQCF, cowpea/soybean, onions, salt, and refined vegetable oil for deep‐frying. The soybean was soaked in water for 15 hr at room temperature and peeled. For the boiled soybean variant, the soybean was boiled in water for 10 min, the water drained, and the boiled grains allowed to cool before dehulling. The boiling duration was shorter than what has been reported by (Kaankuka et al., 1996; Soetan & Oyewole, 2009) because the soybean would still be subjected to further processing (frying) before consumption. More so, a long boiling time may be too time consuming and laborious thus discouraging the adoption of the boiling procedure by household and small‐scale commercial processors; hence the effect of a short heat treatment was investigated. For the cowpea strip variants, the bean grains were soaked in water for 30 min before peeling. 200 g of each of the peeled soybean and cowpea was blended with 50 g of onions using 200 g of water. The resulting paste was then mixed with 200 g of HQCF and 5 g of salt to obtain a soft, non‐sticky dough which was extruded into hot oil and deep fried for 10 min until golden brown. The ratio of mixture was 1:1:1, that is, 200 g of HQCF to 200 g beans/boiled or unboiled soybean to 200 g of water.

### 2.2 Physicochemical and Antinutrient analysis

The three strip samples were analyzed for moisture, protein, fat, ash, crude fiber, total sugars, starch, amylose carbohydrate (by difference), phytate, and tannins.

#### 2.2.1 Moisture content

This was determined using the AOAC (1990) method. The sample was dried at 100–105°C for 24 hr in a draft air Fisher Scientific Isotemp^R^ Oven model 655F. The loss in weight was recorded as moisture content.

#### 2.2.2 Crude fat

This was determined using the AOAC (1990) method in a Soxtec System HT2 fat extractor. Crude fat was extracted from the sample with hexane, and the solvent evaporated off to get the fat. The difference between the initial and final weight of the extraction cup was recorded as the crude fat content.

#### 2.2.3 Ash content

This was determined by the method of AOAC (1990). The method involved burning off moisture and all organic constituents at 600°C in a VULCAN^™^ furnace model 3‐1750. The weight of the residue after incineration was recorded as the Ash content.

#### 2.2.4 Crude protein

This was determined by the Kjeldahl method using Kjeltec^™^ model 2300, as described by Alamu et al. (2016). The method involved digestion of the sample at 420°C for 1 hr to liberate the organically bound nitrogen in the form of ammonium sulfate. The ammonia in the digest (ammonium sulfate) was then distilled off into a boric acid receiver solution, and then titrated with standard Hydrochloric acid. A conversion factor of 6.25 was used to convert from total nitrogen to percentage crude protein.

#### 2.2.5 Starch and sugar

The method of Dubois, Gilles, Hamilton, Rebers, and Smith (1951) was used for the starch and sugar determination. This involved extraction of starch and free sugar from the samples with 95% ethanol, and the hydrolysis of the starch residue with perchloric acid to sugars. The sugar obtained after hydrolysis of the residue was converted to starch by multiplying by 0.9. The absorbance of both starch and sugar was read at 490 nm.

#### 2.2.6 Amylose content

This was determined using the method described by Williams, Wu, Tsai, and Bates (1985). This is a spectrophotometric method based on the formation of deep blue‐colored complex with iodine, the absorbance of which is read at 620 nm.

#### 2.2.7 Crude fiber

This was determined by the Tecator (1978) method using FOSS Fibertec^™^ 2010 model.

#### 2.2.8 Phytate determination

Phytate was determined by a combination of two methods. The extraction and precipitation of phytic acid was done according to the method of Wheeler and Ferrel (1971). Iron in the precipitate was then measured according to the method of Makover (1970). A 4:6 Fe/P atomic ratio was used to calculate the phytic acid content.

#### 2.2.9 Tannin determination

Tannins were determined following the method described by Adegunwa et al. (2011). The reaction is based on the fact that phosphotungstomolybdic acid is reduced by tannin‐like compounds in an alkaline solution, producing a highly colored blue solution which is measured at 760 nm.

### 2.3 Sensory evaluation

In order to compare the sensory ratings between participants who are already familiar with the cowpea variant and participants who are not, the sensory evaluation exercise was conducted among semitrained panellists selected from staff of the International Institute of Tropical Agriculture in Nigeria and Democratic Republic of Congo. The cowpea variant of cassava strips is consumed by participants in Nigeria while the snack itself (both cowpea and soy variant) is relatively new to DRC. A total of 12 panellists took part in the sensory exercise in Nigeria, whereas 18 panellists took part in DRC. Panellists were presented with three coded samples of cassava strips in transparent plastic bags and were asked to rate the degree of liking of the attributes (appearance, aroma, texture, taste, and overall acceptability) of the product on 5‐point hedonic scale where 1 represented ‘dislike very much’ and 5 represented ‘like very much’. The order of sample presentation was randomized in order to prevent positional error. The samples were presented in six different ways and the three panellists received samples in one of the six orders in DRC and while two panellists received samples in one of the six orders in Nigeria, hence a total of 18 panellists.

### 2.4 Consumer evaluation

As the product is relatively new to DRC, a consumer acceptability survey was carried out in this country alone. A total of 148 respondents (88 males and 60 females) evaluated strips made with beans, boiled soybean, and unboiled soybean on 5‐point hedonic scale ranging from dislike very much to like very much. Respondents’ age ranged between 10 and 61 years (average age 28 ± 10) years. Respondents were intercepted at six major market locations in Bukavu, DR Congo. Respondents indicated purchase intent for the three variants of tidbits on 5‐point scale where 1 represented ‘would definitely not buy’ and 5 represented ‘would definitely buy’ and also indicated how much they were willing to pay for a 50 g sample of each tidbit variant in Congolese Francs. Samples were packaged in coded transparent polyethylene bags. The order of sample presentation was also randomized and respondents evaluated each sample one after the other. The samples were presented in six different ways and 25 panellists received samples in one of the six orders, hence a total of 150 panellists less two respondents from making a total of 148 panellists.

### 2.5 Statistical analysis

Data were subjected to Analysis of Variance (ANOVA) with the proximate composition, antinutrient content sensory attributes, consumer preference, and consumption intent being dependent on the type of legume used for the strips (cowpea, boiled soybean, and unboiled soybean). Proximate and antinutrient analyses were carried out in duplicate and the result expressed as mean ± SD. Significance of mean difference was measured at *p *<* *.05 and mean difference was compared using Tukey's HSD. All data were analyzed using SPSS version 20.

## 3 Results

### 3.1 Proximate composition of strip samples

Results of proximate analysis are presented in Table 1. There was significant difference in the proximate composition of the samples. Samples made with cowpea had significantly lower levels of ash, protein, and crude fiber but higher levels of carbohydrate than both the boiled and unboiled soy variant of the snack. On the other hand, boiling for 10 min and draining had a significant effect on the ash, starch, sugar, and amylase contents. Unboiled soy strip samples contained significantly higher ash, sugar, starch, and amylose than the boiled soy variant. Although the boiled soy variant contained higher levels of protein and crude fiber than the unboiled soy variant, this difference was not statistically significant.

**Table 1:** Proximate composition of cassava strips made with boiled soybean, unboiled soybean, and cowpea

Parameter	Boiled soy strips	Unboiled soy strips	Cowpea strips
Moisture (%)	1.53^a^ ± 0.02	1.92^b^ ± 0.04	1.58^a^ ± 0.01
Ash (%)	2.34^a^ ± 0.02	2.80^b^ ±0.01	2.24^a^ ± 0.02
Fat (%)	24.0^a^ ± 0.03	22.63^b^ ± 0.1	23.07^c^ ± 0.02
Protein (%)	9.89^a^ ± 0.37	9.74^a^ ± 0.66	5.58^b^ ± 1.52
Crude fiber (%)	4.21^a^ ± 0.22	3.94^a^ ± 0.11	3.04^b^± 0.08
Carbohydrate (%)	62.25^a^ ± 0.36	62.91^a^ ± .71	67.53^b^ ± 1.52
Sugar (%)	4.70^a^ ± 0.03	5.30^b^ ± 0.12	4.96^a^± 0.02
Starch (%)	57.36^a^ ± 0.00	64.35^b^ ± 0.29	61.31^c^ ± 0.36
Amylose (%)	23.92^a^ ± 0.07	24.78^b^ ± 0.14	25.09^b^ ± 0.00

Means with different superscripts within the same row are statistically different at *p *=* *.05 level.

### 3.2 Antinutrient content of cassava strips

The result of the antinutrient analysis is presented in Table 2. The samples significantly differ in their levels of phytate and tannin. The cowpea variant contained significantly lower levels of antinutrients than both boiled and unboiled soy variants. Results on the effect of boiling on the antinutrient content of the soy variant of the snack showed the significant effect of boiling on the phytate content while no significant effect of boiling was reported for tannin. The boiled soy variant contained 18% less phytate than the unboiled as the phytate level dropped significantly from 5.06 mg/100 g in the unboiled soy variant to 4.29 mg/100 g. Although a drop in tannin content was observed after boiling for 10 min, this difference was not statistically significant.

**Table 2:** Antinutrient contents of cassava strips made with boiled soybean, unboiled soybean, and cowpea

Parameter	Boiled soy strips	Unboiled soy strips	Cowpea strips
Phytate (mg/100 g)	4.29^a^ ± 0.05	5.06^b^ ± 0.1	0.84^c^ ± 0.09
Tannins (mg/100 g)	12.97^a^ ± 0.32	13.03^a^ ± 1.00	4.69^b^ ± 0.16

Means with different superscripts within the same row are statistically different at *p *=* *.05 level.

### 3.3 Sensory evaluation

**Figure 1:**
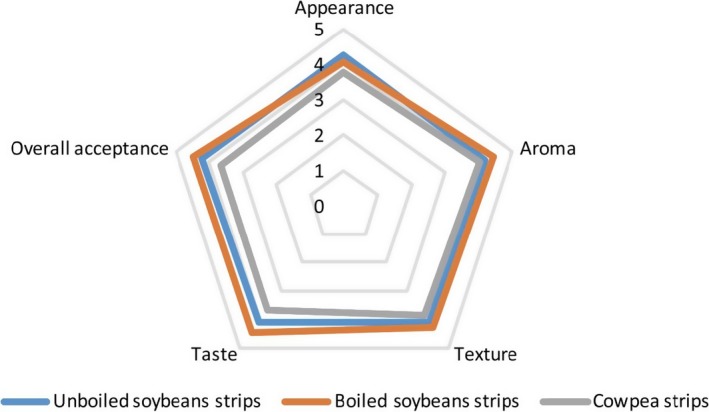
Radar plot of hedonic sensory ratings of unboiled soy strips, boiled soy strips, and cowpea strips in Nigeria

**Figure 2:**
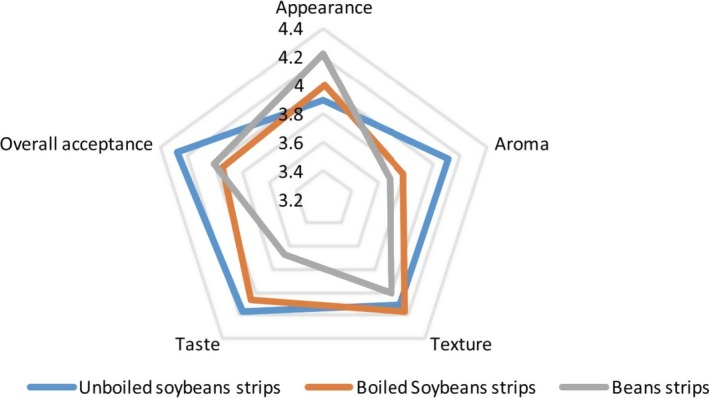
Radar plot of hedonic sensory ratings of unboiled soy strips, boiled soy strips and cowpea strips in DRC

Results of hedonic sensory ratings of the strip samples are presented in Table 3 as well as Figures 1 and 2. Comparing hedonic ratings of sensory attributes between the two countries, results showed that panellists in DR Congo rated the aroma, taste, and overall acceptability of the unboiled soy variant of the strips higher than the boiled soy and bean variant. On the other hand, panellists in Nigeria rated the aroma, taste, texture, and overall acceptance of the boiled soy variant higher than the unboiled soy and bean variant. Nonetheless, the difference in hedonic sensory ratings between the two countries was not statistically significant. While panellists in Nigeria rated the appearance of the unboiled soy variant higher, panellists in DR Congo rated the appearance of the bean variant of the strips higher. Across the two countries, the hedonic ratings were higher for the soy variant (both boiled and unboiled) than for the bean variant.

**Table 3:** Sensory evaluation hedonic ratings for various cassava strips by country

	Nigeria			DR Congo		
	Unboiled soybeans strips	Boiled Soybeans strips	Cowpea strips	Unboiled soybeans strips	Boiled Soybeans strips	Beans/cowpea strips
Appearance	4.25^a^ ± 0.45	4.08^a^ ± 0.51	3.75^a^ ± 0.75	3.89^a^ ± 0.96	4.00^a^ ± 0.91	4.22^a^ ± 0.88
Aroma	4.17^a^ ± 0.39	4.42^a^ ± 0.51	4.00^a^ ± 0.74	4.11^a^ ± 0.68	3.78^a^ ± 1.21	3.68^a^ ± 1.14
Texture	4.08^a^ ± 0.29	4.25^a^ ± 0.62	3.83^a^ ± 1.03	4.11^a^ ± 0.90	4.17^a^ ± 0.79	4.00^a^ ± 0.84
Taste	4.08^a,b^±0.29	4.42^a^ ± 0.51	3.67^b^ ± 0.78	4.17^a^ ± 0.79	4.06^a^ ± 0.87	3.67^b^ ± 1.03
Overall acceptance	4.25^a^ ± 0.45	4.50^a^ ± 0.52	3.67^b^ ± 0.78	4.28^a^ ± 0.67	3.94^a^ ± 0.94	4.00^a^ ± 1.00

Means with different superscripts within the same row are statistically different at *p *=* *.05 level.

### 3.4 Consumer evaluation

The results of the consumer survey also show no significant difference (*p *>* *.05) in mean hedonic ratings of the attributes of the three strip samples. Although similar to the results of the sensory evaluation, participants in the consumer survey rated the aroma of the unboiled soy variant higher than that of the bean and boiled soy variant, however, the difference was not statistically significant. Also, no significant difference in purchase intent and willingness to pay was reported among the three samples. Results are presented in Table 4.

**Table 4:** Sensory hedonic ratings, purchase intent, and WTP for cassava strips made with unboiled soybean, boiled soybean, and bean

Attributes	Unboiled soybean strips	Boiled soybean strips	Bean/cowpea strips
Appearance	4.42 ± 0.87^a^	4.41 ± 0.84	4.57 ± 0.70
Aroma	4.32 ± 0.93	4.28 ± 0.80	4.23 ± 0.96
Texture	4.10 ± 1.04	4.10 ± 1.01	4.02 ± 1.14
Taste	4.22 ± 1.04	4.35 ± 0.88	4.17 ± 0.98
Purchase intent	4.19 ± 1.07	4.29 ± 0.86	4.14 ± 1.05
Willingness to pay (France)	238.70 ± 214.22	247.22 ± 276.14	248.8 ± 185.26

a Mean ± SE.

## 4 Discussion

The significantly lower starch content of the boiled soy variant of the strips when compared to the unboiled soy variant may be due to leaching of the starch into the boiling water during boiling and draining. During wet heat treatment there is a considerable loss of low molecular weight carbohydrates (i.e., mono‐ and disaccharides) into the processing water (Nyman, Palsson, & Asp, 1987). A similar explanation can be given for the lower ash content of the boiled soy variant when compared to the unboiled. These results are consistent with account of Esenwah and Ikenebomeh (2008) who reported a significant drop in ash and carbohydrate content of locust beans after soaking and boiling due to leaching of soluble organic salts into processing water during boiling. This result also corroborated that of Ramadan (2012) who reported a significant drop in ash content of soybean after soaking and cooking. Also, the significantly lower sugar content of the soy variant of the strips when compared with the bean variant may be to some extent attributable to leaching of the sugar during soaking considering the two soy variants were soaked in water for 15 hr while the bean variant was soaked for only 30 min.

Both boiled and unboiled soy variants of the cassava strips contained significantly higher levels of protein than the cowpea variant. This is no surprise as raw soybean grains have a higher protein content than beans and other legumes. Ramadan (2012) reported a protein content of 35.55% and 37.50% in Giza 21 and Giza 35 varieties of soybean, respectively. Mark (1999) also stated a protein content of 38% in some other soybean varieties, whereas cowpea contains between 22% and 28% protein. Contrary to the significant drop in ash, starch, sugar, and amylose content of the soy variant due to boiling, the reverse was observed for protein and crude fiber. Boiling resulted in a slight increase in protein and crude fiber content; nonetheless, the difference was not statistically significant. This was similar to the result obtained by Ramadan (2012) in his study to investigate the effect of soaking and cooking on chemical composition of two soybean varieties. Ramadan (2012) reported a rise in protein content from 35.55% to 38.55% and from 37.50 to 40.25% for Giza 21 and Giza 35 varieties of soybean, respectively. He also reported an increase in crude fiber content for the same soy bean varieties from 7.12% to 9.85% and 9.30% to 11.95%, respectively. The significantly higher loss of sugar as against proteins reiterates the report of Huma, Anjum, Sehar, Khan, and Hussain (2008) which was attributed to the solubility of sugars in water. The inconsequential increase in protein content in the boiled soy variant as against the reduction reported by (Huma et al., 2008) may be as a result of the short boiling duration which was not long enough to allow chemical degradation of proteins into water soluble amino acids.

The results of sensory evaluation among panellists in Nigeria and DR Congo showed cross‐cultural differences in preference for the attributes of the cassava strips based on the legume type and preprocessing procedure the legume was subjected to. Although panellists in both countries preferred most of attributes of the soy variants to the cowpea variants, panellists in DRCongo seemed to favor the beany aroma and taste of the unboiled soy variant while panellists in Nigeria preferred the taste and aroma of the boiled soy variant though not significantly more than the unboiled.

All three sources of protein used in the formulation gave finished products with acceptable sensory properties although soybean gave a finished product with more acceptable sensory attributes among panellists in the two countries where the sensory evaluation exercise was conducted. Hence, it can be concluded that soybean gives the best product in terms of nutritional quality and sensory acceptance, whereas the bean variant has the advantage of lower antinutrients than both boiled and unboiled soybean. As boiling resulted in only 18% reduction in the phytate level with not much impact on protein content and sensory acceptability, the preboiling step can be skipped without serious consequences on the nutritional value and sensory properties.

## 5 Conclusion

This study not only demonstrated the potential of HQCF together with two protein‐rich grain legumes as ingredients in the production of snack food in low income populations where cassava is widely grown, it also identified the nutritional and antinutritional factors to be considered in choosing legumes. The outcome of this study showed that each legume type used as well as the preprocessing procedure has its pro‐ and con‐nutritional benefits. Using cowpea in the formulation resulted in a finished product with low protein content while compared with soybean, nonetheless the significantly lower antinutrient content made up for the low protein content, whereas the reverse was the case for the formulation containing soybean. However, phytate and tannin levels of all three variants of the snack in this study fell within the permissible level. Ndidi et al. (2014) reported a permissible level of 20 mg/100 g and 250–500 mg/100 g for tannins and phytate, respectively. Also, while a short boiling time significantly reduced the level of one of the antinutrients and also increased the protein and crude fiber content, boiling also resulted in leaching of other soluble organic salts such as ash, sugar, and starch. The choice of protein‐rich legume used in the snack formulation also depends on other factors such as price and availability of these legumes, among other things. This innovation, if adopted, will help to improve the livelihoods of poor farmers in developing countries growing cassava and legumes and also contribute to alleviate protein‐malnutrition in these countries.

## Conflict of Interest

The authors have declared no conflict of interest.
